# Attitudes and Perceptions amongst Critical Care Physicians towards Handshake Antimicrobial Stewardship Rounds

**DOI:** 10.7759/cureus.6419

**Published:** 2019-12-19

**Authors:** Bryan Evans, Justin Kosar, Shaqil Peermohamed

**Affiliations:** 1 Internal Medicine, University of Saskatchewan College of Medicine, Saskatoon, CAN; 2 Miscellaneous, Saskatchewan Health Authority, Saskatoon, CAN; 3 Internal Medicine / Infectious Disease, University of Saskatchewan College of Medicine, Saskatoon, CAN

**Keywords:** handshake stewardship, antimicrobial stewardship, critical care, attitudes, perceptions, survey

## Abstract

Rationale

In an era of antimicrobial resistance, antimicrobial stewardship programs are tasked with reducing inappropriate use of antimicrobials in community and hospital settings. Intensive care units are unique, high-stakes environments where high usage of broad-spectrum antimicrobials is often seen. Handshake stewardship has emerged as an effective mode of prospective audit and feedback to help optimize antimicrobial usage, emphasizing an in-person approach to providing feedback.

Objectives

Six months following the implementation of handshake stewardship rounds in our intensive care unit, we performed a cross-sectional survey of critical care physicians to assess their attitudes and perceptions towards handshake stewardship rounds and preferred mode of delivery of antimicrobial stewardship prospective audit and feedback strategies.

Methods

A web-based survey was distributed to 22 critical care physicians working in our hospital and responses were collected over a two-week period.

Measurements and Main Results

Most critical care physicians believe that handshake stewardship rounds improve the quality of patient care (85.7%) and few believe that handshake stewardship rounds are an ineffective use of their time (14.3%). The majority of critical care physicians believe formal, scheduled rounds with face-to-face verbal interaction are very useful compared to providing written suggestions in the absence of face-to-face interaction (71.4% vs 0%).

Conclusions

Based upon our survey results, handshake stewardship is valued amongst the majority of critical care physicians. Antimicrobial stewardship prospective audit and feedback strategies emphasizing face-to-face interaction are favored amongst critical care physicians.

## Introduction

Antibiotic stewardship has been defined as the “coordinated interventions designed to improve and measure the appropriate use of antibiotic agents by promoting the selection of the optimal antibiotic drug regimen including dosing, duration of therapy, and route of administration” [1]. Prior studies have shown that approximately 50% of antimicrobial usage is unnecessary or inappropriate in hospital, community and ambulatory settings [2-5]. Implementing effective antimicrobial stewardship strategies in intensive care units is of particular relevance, given the high density of antimicrobial use in these hospital settings [6-8]. Implementation of antimicrobial stewardship programs in intensive care units has been shown to reduce broad-spectrum antimicrobial utilization, decrease the incidence of infections and colonization with multi-drug resistant organisms and reduce antimicrobial related adverse events [9-12]. A prior Canadian survey showed that critical care physicians feel antimicrobial stewardship programs provide a valuable service of benefit to both patients and physicians [13].

Several barriers may impede the success of antimicrobial stewardship interventions in intensive care units, such as diagnostic uncertainty and fear of not adequately treating potential pathogens [14-16]. Prospective audit and feedback and pre-authorization have been recognized as core strategies of an antimicrobial stewardship program; however, the latter may have unintended consequences as a restrictive strategy including delays in time to first antibiotic dose and negative systemic effects on inter-professional collaboration [1,17]. Alternatively, prospective audit and feedback strategies may be more suitable in critical care environments if they increase capacity and engagement with prescribers [7,17]. Handshake stewardship has emerged as an extended derivative of prospective audit and feedback that places emphasis on three main components: lack of restriction and preauthorization, pharmacist-physician review of all antimicrobials and a rounding-based, in-person approach to feedback carried out by a pharmacist-physician team [18-19].

Six months following the implementation of handshake stewardship rounds occurring three times weekly in our medical-surgical critical care unit, we performed a cross-sectional survey amongst critical care physicians in our center to determine their attitudes and perceptions towards handshake stewardship rounds.

## Materials and methods

A web-based cross-sectional survey was designed and distributed to all critical care physicians working in Saskatoon, Saskatchewan, Canada, six months following the implementation of handshake stewardship rounds in our center.

The generation of items for the survey tool was developed in collaboration with experts in infectious diseases, critical care and antimicrobial stewardship. The questions were designed to assess the attitudes and perceptions of antimicrobial stewardship. Survey items assessed attitudes, perceptions and experiences towards handshake stewardship rounds using a five-point Likert-type scale with responses grouped as strongly agree/agree, neutral, and disagree/strongly disagree.

The introductory email and link to the online survey were sent in May 2017 using SurveyMonkey (SurveyMonkey Inc.®; Palo Alto, CA, USA) and were distributed to all identified critical care physicians, followed by two reminder emails sent every three days. The introductory email included a description of the purpose of the survey and the value of providing anonymous feedback. Survey responses were collected over a period of two weeks. The research proposal and survey tool were approved by the University of Saskatchewan Research Ethics Board.

## Results

The survey was distributed to 22 critical care physicians who worked in Saskatoon, Saskatchewan, Canada at the time of the survey, with a response rate of 68.2% (n = 15/22). One respondent reported no longer working in critical care so was excluded from our analysis; all 14 respondents included in our analysis reported participating in handshake stewardship rounds in the prior six months. Most respondents (85.7%) who had participated in handshake stewardship rounds reported that these rounds improve the quality of patient care. In addition, the minority of respondents (14.3%) disagreed that handshake stewardship rounds were an effective use of their time.

More than half of our respondents (57.1%) reported that handshake stewardship rounds changed their use of antimicrobials in critical care. Amongst these eight individuals reporting a change in the use of antimicrobials, the most commonly reported perceived changes in antimicrobial prescribing behaviors included using more targeted therapy (100%) and using a shorter duration of therapy (50%). Interestingly, 42.8% of survey respondents reported that handshake stewardship rounds resulted in greater discussion of antimicrobial prescribing during their daily morning bedside rounds. Furthermore, most survey respondents (85.7%) disagreed that handshake stewardship rounds impeded upon their autonomy as a critical care physician.

**Figure 1:**
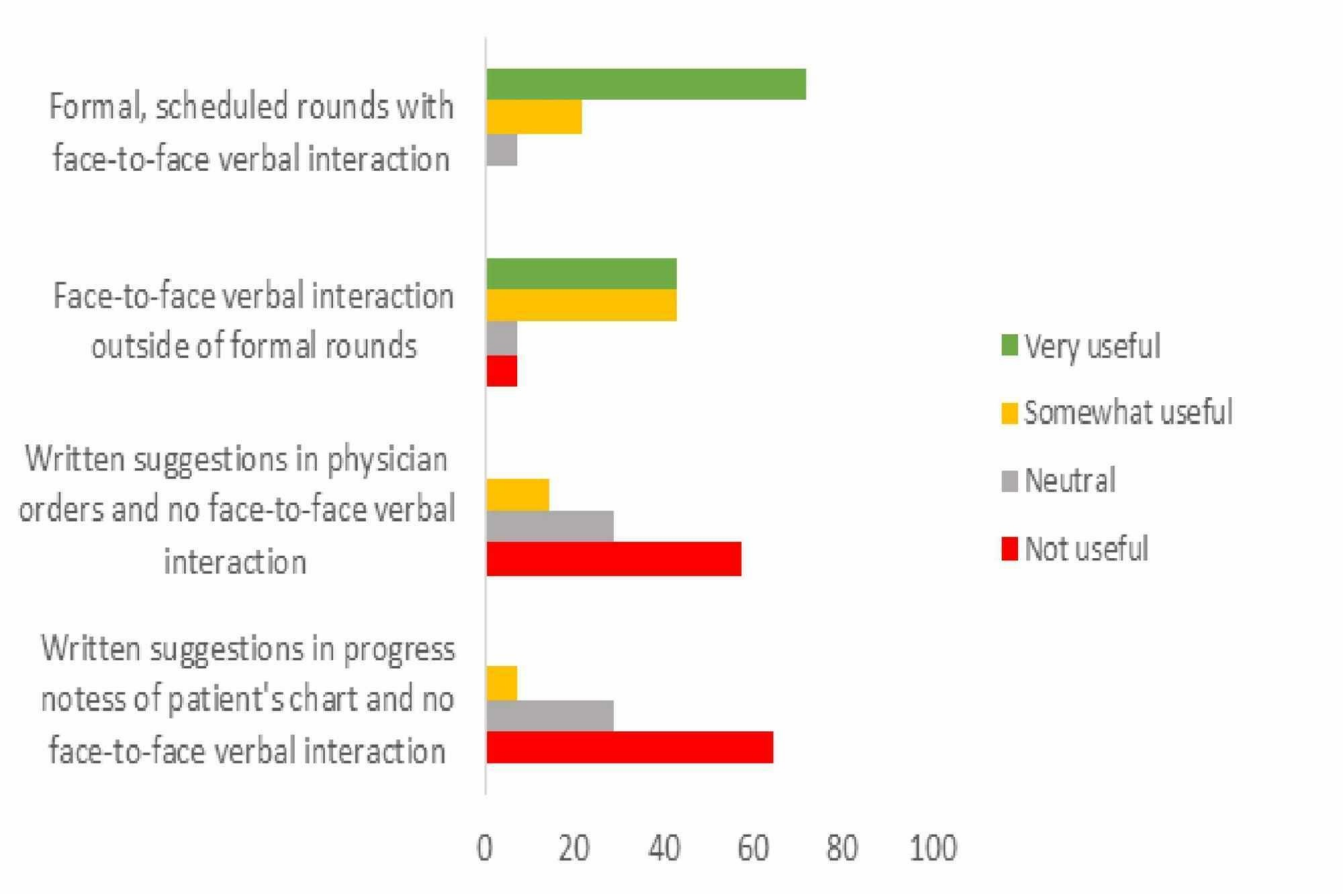
Reported utility of different models of prospective audit and feedback amongst critical care physicians (n = 14)

Critical care physicians were also asked to rate the perceived utility of different modes of delivering prospective audit and feedback using a four-point Likert-type scale of very useful, somewhat useful, neutral and not useful. The four modalities presented included formal scheduled rounds with face-to-face interaction, face-to-face interaction but not formally scheduled rounds, written suggestions in practitioner orders without face-to-face interaction, and lastly, written suggestions in patient chart progress notes without face-to-face interaction. Formal scheduled rounds with face-to-face verbal interaction were rated as being very useful and the preferred modality amongst critical care physicians (71.4%), followed by face-to-face verbal interaction outside of formal rounds (42.9%, Figure 1). Importantly, most respondents believed that written suggestions in physician orders or progress notes without face-to-face verbal interaction would not be useful (64.3% and 57.1%, respectively, Figure 1).

## Discussion

Handshake stewardship rounds are valued and supported amongst most critical care physicians as a form of prospective audit and feedback that improves patient quality of care. In addition, most critical care physicians believe handshake stewardship rounds are an effective use of time and do not impede upon physician autonomy. While many critical care physicians self-reported that handshake stewardship rounds changed their antimicrobial prescribing behaviors and resulted in greater discussion of antimicrobials during their morning rounds, further studies are needed to assess the impact of handshake stewardship rounds on the appropriateness of antimicrobial use and other pertinent clinical outcomes in critical care settings. Our survey supports current literature that the preferred delivery model of prospective audit and feedback amongst critical care physicians involves face-to-face interaction [13]. This reinforces the importance of approaches that foster collaboration, convey trust and optimize communication amongst key stakeholders [18-19]. 

Our survey has several limitations. Firstly, it is possible that our survey may not have captured responses of all critical care physicians who had participated in handshake stewardship rounds. However, our survey response rate of 68.2% is higher than average email response rates amongst physicians, increasing the likelihood that our survey responses captured feedback from most of our critical care physicians who had participated in handshake stewardship rounds [20]. In addition, deploying our survey six months following the implementation of handshake stewardship rounds ensured all critical care physicians at our site had participated in handshake stewardship rounds at least once. Secondly, survey responses do not reflect the frequency in which a critical care physician may have participated in handshake stewardship rounds; it is possible that more frequent participation in handshake stewardship rounds could affect their attitudes and perceptions towards handshake stewardship and influence their antimicrobial prescribing behavior. It is possible that more frequent participation in handshake stewardship rounds may have led to greater discussion and reflection on antibiotics during critical care rounds and altered prescribing behaviors. Thirdly, respondent bias could also impact our results such that critical care physicians who feel most strongly, either positively or negatively, about handshake stewardship rounds may have been more likely to respond. Finally, implementation of handshake stewardship requires physicians and pharmacists with background training and expertise in infectious diseases and antimicrobial stewardship as well as engagement from critical care physicians and pharmacists; such a strategy may not be feasible in all centers especially those with limited antimicrobial stewardship program personnel resources.

## Conclusions

Handshake stewardship is valued as a mode of prospective audit and feedback amongst critical care physicians. Prospective audit and feedback strategies emphasizing face-to-face interaction and bi-directional, verbal feedback may be of greater value compared to other forms of delivering prospective audit and feedback in high-stakes environments such as critical care.
